# Books and Authors

**Published:** 1905-12

**Authors:** 


### BOOKS AND AUTHORS.

**The Surgical Assistant.** A Manual for Students, Practitioners, Hospital Internes and Nurses. By **Walter M. Brickner**, B.S., M.D., Assistant Surgeon, Mount Sinai Hospital, Out-Patient Department. Three hundred and sixty pages, 123 original illustrations and 116 illustrations of surgical instruments. New York: The International Journal of Surgery Co. 1905. (Price, $2.00 net.)

The material that goes to make up this book is to be found in no other single volume. Here and there, scattered through textbooks and monographs, are allusions to the duties of assistants in the operating rooms of surgeons, but in this volume the topic is dealt with in detail and a completeness that makes it stand unrivaled in the field of surgical lectures.

The chapter on the anesthetist is to be commended especially, and should be read by every person who has to do with operations,—surgeon, anesthetist, assistant and nurses.

It is a book, considered from every viewpoint, that cannot fail to do much good. It has a practical value for every person engaged in the practice of medicine as well as for those immediately concerned in hospital work. The illustrations are many and useful, though some of the half-tones could and should be made clearer. On the whole it is a book that justifies distinct praise.

---

**The Treatment of Fractures.** With notes upon a Few Common Dislocations. By **Charles L. Scudder**, M.D., Surgeon to the Massachusetts General Hospital. Fifth edition, revised and enlarged. Octavo, 563 pages, with 739 original illustrations. Philadelphia and London: W. B. Saunders & Co. 1905. (Polished buckram, $5.00 net; half-morocco, $6.00 net.)

The successive editions of Scudder's treatise follow at such short intervals that the reviewer becomes amazed at the industry of the author and enterprise of the publishers.

The first edition appeared in 1900 and each year since that time a new issue has been required to meet the great demand for the book. This fact has enabled the author to keep its teachings constantly in touch with the latest knowledge.

In the present edition the text has been revised, new material has been added, and many new illustrations have been inserted, increasing the latter by fifty,— many of which are *x*-ray plates illustrating the actual line of fracture.

It is difficult to understand how the work could be improved or why everyone who aspires to be a surgeon should not possess it.

---

**Treatise on Orthopedic Surgery.** By **Edward H. Bradford,** M.D., Professor of Orthopedic Surgery, Harvard Medical School. And **Robert W. Lovett,** M.D., Assistant in Orthopedic Surgery, Harvard Medical School. Third edition. Octavo, pp. vi.-669. Illustrated by 592 engravings. New York: William Wood & Co. 1905. (Price: cloth, $5.00; sheep, $5.75, net.)

It is surprising how rapidly advances are making in orthopedics. A comparison of this edition with the second published in 1899, has impressed this fact upon us with great force. It then seemed that almost the last word had been said on the subject; but this edition is almost a new treatise, so much being either quite new, or else recast to adapt it to present conditions and methods.

For some years Bradford and Lovett have been accepted as authorities on questions relating to orthopedic surgery and treatment; the treatise written by them occupies the front rank in the literature of prevention and correction of deformities of the human body; and their methods have received the approval of the professional world.

The chapter relating to congenital dislocation of the hip and its treatment is the most complete yet published and deserves great praise. The illustrations of this subject are superb and make clear the descriptions of methods of reduction and dressing.

Talipes is another topic that is masterfully handled. It is to be hoped that no person will be allowed to pass the period of infancy with club foot; it is imperative upon parents and guardians that this unsightly deformity be corrected while yet it can be completely remedied. Flat-foot and other deformities of the feet make an interesting chapter and one, too, of great importance.

The number and quality of the illustrations in this edition have been greatly increased, many of which are original, and all are excellent. As a textbook, as a guide in practice, as a book of reference, and as a complete treatise on orthopedic surgery the work has no superior.

---

**Saunders's Pocket Medical Formulary.** By **William M. Powell,** M.D., author of "Essentials of Diseases of Children;" containing 1831

formulas from the best known authorities. With an Appendix containing Posological Table, Formulas and Doses for Hypodermic Medication, Poisons and their Antidotes, etc., etc. Seventh edition, revised. Philadelphia and London: W. B. Saunders & Co. 1905. (In flexible morocco, with side index, wallet, and flap, $1.75 net.)

In revising this book the author has dropped formulas that have become obsolete, and added more than four hundred and sixty new ones, thus making it one of the most complete compilations of its class on the market. Great care has been taken in selecting the material from approved sources, and many of the newer remedies have been given in some of the formulas. The book is well interleaved with blank pages for such additions as may be desired, making a practical and useful aid to the younger clinician.

---

**Theraputics: Its Principles and Practice.** By **Horatio C. Wood,** M.D., Professor of Materia Medica and Theraputics in the University of Pennsylvania. Twelfth edition revised and adapted to the Eighth (1905) edition of the United States Pharmacopeia, by Horatio C. Wood, and Horatio C. Wood, Jr., M.D., demonstrator of pharmacodynamics in the University of Pennsylvania. Philadelphia and London: J. B. Lippincott Company. 1905.

From the year 1875, when the first edition of this famous work was issued, to 1905 which witnesses its twelfth output is a far cry. Few medical books can point to thirty years of continued favor with the profession, and at the same time to have kept pace with the foremost progress in medicine. Wood, the father, has witnessed this phenominal fact with reference to the work of his head, and heart, and hand; and now comes forward the son to assist in continuing the revisions. Who knows but that thirty years more may be added to the record and prosperity of "Wood's Theraputics!" Let us hope so!

The publication of this edition has been delayed for some two years awaiting the appearance of the eighth decennial revision of the United States Pharmacopeia, in order that it might conform to the latter. The 1900 edition of the pharmacopeia, which but lately has been published, contains so many radical changes that the additions to Wood's twelfth edition are in excess of any previous publication of the book.

The editors themselves, too, have found many alterations necessary, independently of those called for by the pharmacopeia. Many chapters have been rearranged, others rewritten, and some added, which makes a complete book of reference containing the very latest information. As indicating the increase in the theraputic armament it may be mentioned that over seventy new drugs have been added to those described in former editions. The labor necessary to keep the treatise abreast of the period is enormous but it has been faithfully and judiciously expended.

